# Primary amoebic meningoencephalitis caused by *Naegleria fowleri* in a 6-year-old girl: case report

**DOI:** 10.3389/fped.2026.1801355

**Published:** 2026-04-10

**Authors:** Lihua Fu, Yu Zhang, Lixiang Wang, Xiaoling Li

**Affiliations:** 1 Department of Pediatrics, Taihe Hospital of Chinese Medicine Affiliated to Anhui University of Chinese Medicine, Fuyang, China; 2 Department of Rehabilitation, Taihe Hospital of Chinese Medicine Affiliated to Anhui University of Chinese Medicine, Fuyang, China

**Keywords:** cerebrospinal fluid, metagenomic next-generation sequencing, *Naegleria fowleri*, pediatric, primary amoebic meningoencephalitis

## Abstract

**Background:**

Primary amoebic meningoencephalitis (PAM) is caused by *Naegleria fowleri*, a rare but highly fatal central nervous system infection with a mortality rate exceeding 95%. Early diagnosis is challenging due to the close similarity of its clinical manifestations and cerebrospinal fluid (CSF) findings to those of acute bacterial meningitis. Metagenomic next-generation sequencing (mNGS) has become a vital tool for identifying rare or unexpected pathogens.

**Case presentation:**

A previously healthy 6-year-old girl was admitted with fever, vomiting, and headache of 1 day’s duration. Six days before symptom onset, she had played in natural freshwater bodies. After admission, she developed persistent high fever and rapidly progressive altered mental status, followed by two episodes of generalized tonic-clonic seizures, hemoptysis, acute respiratory failure, and circulatory shock. Initial cranial magnetic resonance imaging showed no abnormalities. CSF analysis revealed marked inflammatory changes: a white blood cell count of 3,072 × 10^6^/L, markedly elevated protein (3,667.6 mg/L), and significantly decreased glucose (0.08 mmol/L). Despite administration of broad-spectrum antibiotics, glucocorticoids, osmotherapy, and comprehensive intensive care unit management, the patient died approximately 11 h after admission following three cardiac arrests. Two days postmortem, CSF mNGS confirmed infection with *Naegleria fowleri* (copy number 3 × 10^5^ copies/mL), establishing the diagnosis of PAM.

**Conclusions:**

This pediatric case serves as a warning that PAM should be considered in children with a history of freshwater exposure and rapidly progressive meningoencephalitis, even when early imaging is normal and CSF findings resemble bacterial meningitis. Early lumbar puncture, rapid molecular diagnostics, and heightened clinician vigilance are critical for the timely initiation of targeted therapy.

## Introduction

Primary amoebic meningoencephalitis (PAM) is an acute, hemorrhagic, necrotizing meningoencephalitis caused by the free-living amoeba *Naegleria fowleri* (1, 2). Clinical progression is extremely rapid, with death typically occurring within days of symptom onset. This amoeba is widely distributed in warm freshwater environments, including lakes, rivers, hot springs, artificial water bodies, and inadequately disinfected swimming pools and water supply systems (3). The pathogen typically enters through the nasal cavity during exposure to contaminated water, penetrating the olfactory epithelium and cribriform plate to reach the central nervous system (CNS), thereby triggering fulminant meningoencephalitis.

A global literature review indicates that since the first reported case in the 1960s, approximately 400–500 cases of PAM have been reported worldwide, though the actual incidence may be higher (2, 3). The case fatality rate consistently exceeds 95%, with only a handful of survivors reported. These survivors typically received definitive diagnosis at the earliest possible stage, followed by intensive combined anti-amoebic therapy and aggressive intracranial pressure management (3, 4). Due to its acute onset, rapid progression, and early symptoms (fever, headache, vomiting, neck stiffness, altered consciousness, etc.) that closely mimic acute bacterial meningitis, PAM is frequently misdiagnosed as bacterial or viral meningoencephalitis. Meanwhile, routine cerebrospinal fluid (CSF) bacterial smears and cultures often yield negative results, further complicating early recognition (1, 2).

In recent years, multiple reviews and case series have highlighted that the clinical, laboratory, and imaging manifestations of PAM are particularly severe in pediatric populations, with most cases having a clear freshwater exposure history (2, 4–6). China and other countries have reported multiple pediatric PAM cases, including children with fulminant meningoencephalitis confirmed by CSF and blood metagenomic next-generation sequencing (mNGS), as well as cases associated with exposures such as indoor swimming pools, religious nasal rinsing, and nasal irrigation (7–10). As a non-targeted pathogen detection technology, mNGS is particularly suitable for rapidly progressing central nervous system infections with negative conventional tests or high diagnostic uncertainty. It has provided key etiologic evidence in multiple PAM cases (8–12).

This report describes a case of PAM in a 6-year-old girl who presented with sudden onset and rapid progression, succumbing to the disease approximately 11 h after admission. The diagnosis of *Naegleria fowleri* infection was ultimately confirmed postmortem via CSF mNGS. We discuss the clinical features, diagnostic process, and implications for prevention and control based on the literature, aiming to enhance clinicians' awareness of this rare yet fatal infection (Table 1).

**Table 1:** Timeline of key clinical events and investigations.

Time	Key clinical manifestations	Key investigations and results	Clinical impression
Pre-admission (Sep 2, 2025)	Fever, vomiting, headache for 1 day	Outside labs: WBC 10.40 × 10^9^/L, neutrophils 83.2% (8.65 × 10^9^/L), lymphocytes 9.6% (1.00 × 10^9^/L), Hb 130 g/L, PLT 210 × 10^9^/L, hs-CRP 34.22 mg/L	Early nonspecific presentation
Sep 3, 07:20	Persistent fever, recurrent vomiting, lethargy; T 39.5 °C; tachycardia/tachypnea; BP stable	Serum PCT 0.46 ng/mL	Suspected infection; admitted
Sep 3, 10:00	Rapidly progressive somnolence; difficult arousal	Brain MRI (non-contrast): normal; EEG/brain topographic mapping: normal	CNS infection suspected despite normal early imaging
Sep 3, 10:40	First generalized tonic–clonic seizure (3 min)	Lumbar puncture performed; CSF sent for routine analysis and mNGS	Rapid neurological deterioration
Sep 3, 11:20	Second generalized tonic–clonic seizure (3 min)	-	Respiratory deterioration soon after
Sep 3, 11:18	Acute respiratory failure	ABG (pre-intubation): pH 7.378, PCO_2_ 27.4 mmHg, PO_2_ 39 mmHg, BE −9.7 mmol/L, SO_2_ 73%, lactate 2.50 mmol/L	Severe hypoxemia with metabolic acidosis
Sep 3, PICU admission	Coma; severe respiratory distress; cyanosis; anisocoria (L 3.0 mm, R 2.0 mm)	Vitals: T 39.5 °C, HR 183/min, RR 43/min, BP 104/79 mmHg, SpO_2_ 83%	Fulminant meningoencephalitis with systemic failure
Sep 3, 12:35	-	CSF: WBC 3,072 × 10^6^/L; RBC 2.00 × 10^9^/L; Pandy test positive; protein 3,667.6 mg/L; glucose 0.08 mmol/L	CSF profile mimicking severe purulent meningitis
Sep 3, 13:39	Worsening respiratory failure	Chest x-ray: extensive bilateral infiltrates; focal right lung consolidation	Severe lung injury/ARDS-like changes
Sep 3, afternoon	Refractory shock; coagulopathy	PCT 15.18 ng/mL; PT 16.90 s; INR 1.34; PTA 64.81%; fibrinogen 6.93 g/L; D-dimer 15.05 mg/L	Severe systemic inflammatory response
Sep 3, 15:19/15:56/17:41	Three cardiac arrests; CPR performed	Ventricular fibrillation during final arrest; defibrillation (40 J); no sustained ROSC	Death 11 h after admission
Sep 5	-	CSF mNGS detected *Naegleria fowleri*; 3 × 10^5^ copies/mL, high signal strength	Definitive diagnosis of PAM

WBC, white blood cell count; Hb, hemoglobin; PLT, platelet count; hs-CRP, high-sensitivity C-reactive protein; PCT, procalcitonin; MRI, magnetic resonance imaging; EEG, electroencephalography; CSF, cerebrospinal fluid; mNGS, metagenomic next-generation sequencing; ABG, arterial blood gas; PCO_2_, partial pressure of carbon dioxide; PO_2_, partial pressure of oxygen; BE, base excess; SO_2_, oxygen saturation; PICU, pediatric intensive care unit; HR, heart rate; RR, respiratory rate; BP, blood pressure; SpO_2_, peripheral oxygen saturation; PT, prothrombin time; INR, international normalized ratio; PTA, prothrombin activity; ARDS, acute respiratory distress syndrome; CPR, cardiopulmonary resuscitation; ROSC, return of spontaneous circulation; PAM, primary amoebic meningoencephalitis.

## Case timeline

**Figure 1:**
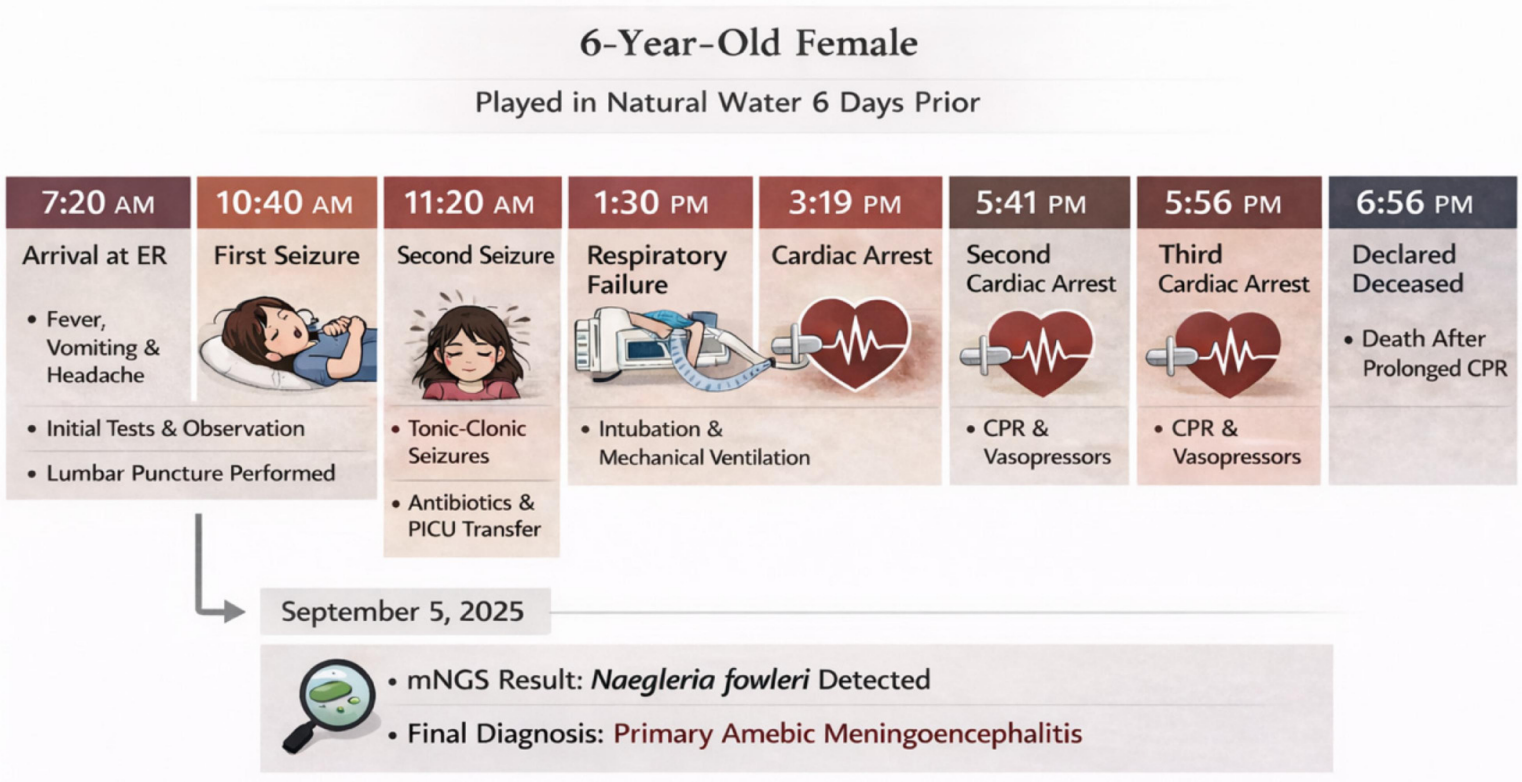
Clinical timeline of a 6-year-old girl with primary amebic meningoencephalitis.

A schematic timeline summarizing the key clinical events from hospital admission to death is presented in Figure 1.

## Case presentation

The patient was a previously healthy 6-year-old girl with normal growth and development. She had no history of chronic underlying disease or immunodeficiency, and there was no family history of neurological or infectious disease. On September 3, 2025, at 07:20, accompanied by family members, she presented to our emergency department with a 1-day history of fever and vomiting for 1 day, accompanied by headache. According to the family, the patient had played in natural freshwater bodies near her home, and the last known freshwater exposure occurred approximately 6 days before the onset of symptoms. Specific details regarding water temperature, water source type, nasal water entry, or submersion activities could not be reliably confirmed. The patient had no recent history of travel. Symptoms progressed rapidly, and the patient died approximately 35 h after symptom onset.

Upon admission, the patient remained febrile with recurrent vomiting, exhibiting lethargy and sluggish responsiveness. Physical examination revealed a temperature of 39.5 °C, tachycardia and tachypnea, stable blood pressure, no significant neck stiffness or opisthotonos, and no characteristic rash of the skin or mucous membranes. No specific focal neurological deficits were documented at that time. Laboratory tests conducted at a local health center one day prior to admission showed: white blood cell count 10.40 × 10^9^/L, neutrophils 8.65 × 10^9^/L (83.2%), lymphocytes 1.00 × 10^9^/L (9.6%), hemoglobin 130 g/L, platelets 210 × 10^9^/L, and high-sensitivity C-reactive protein 34.22 mg/L. An upright abdominal plain radiograph (digital radiography) showed no evidence of intestinal perforation or obstruction. Cranial magnetic resonance imaging (MRI) and electroencephalogram performed on the day of admission showed no significant abnormalities. Serum procalcitonin (PCT) was 0.46 ng/mL at admission. The emergency department made a preliminary diagnosis of fever of unknown origin and admitted the patient for further observation and treatment.

Following admission, intravenous cefotaxime sodium was continued for anti-infective therapy, along with an intravenous bolus of mannitol to reduce intracranial pressure. Concurrently, the patient received continuous electrocardiogram (ECG) monitoring, supplemental oxygen, and symptomatic supportive care. Around 10:00, the patient developed impaired consciousness, presenting with worsening somnolence, markedly blunted responses, and progressively difficult arousal. Given persistent high fever, headache, vomiting, and altered consciousness, central nervous system infection was strongly suspected. After ruling out obvious signs of intracranial mass effect, an emergency lumbar puncture was performed. CSF samples were sent for routine analysis, cytology, biochemistry, and metagenomic next-generation sequencing.

At approximately 10:40, the patient experienced the first generalized tonic-clonic seizure, presenting with loss of consciousness, limb convulsions, and foaming at the mouth. There was no incontinence of urine or stool. The episode lasted approximately 3 min. Chloral hydrate enema was administered for sedation, and intravenous infusion of methylprednisolone sodium succinate was initiated to alleviate systemic inflammatory response. Concurrently, antibiotics were escalated to intravenous ceftriaxone sodium. Around 11:20, the patient experienced another similar generalized convulsive episode lasting approximately 3 min. Symptomatic management with intravenous phenobarbital, fluid replacement, and antipyretic therapy resulted in temporary symptom relief.

Following the second seizure, the patient developed significant coughing and hemoptysis, with oxygen saturation rapidly dropping to approximately 80%. Diffuse crackles were audible in both lungs, raising suspicion of acute pulmonary hemorrhage and acute respiratory failure. Immediate mask oxygen therapy was initiated, and the patient was urgently transferred to the Pediatric Intensive Care Unit (PICU).

Arterial blood gas analysis before and after PICU admission showed the following: 11:18 (pre-intubation): pH 7.378, partial pressure of carbon dioxide (PCO_2_) 27.4 mmHg, partial pressure of oxygen (PO_2_) 39 mmHg, base excess (BE) −9.7 mmol/L, oxygen saturation 73%, blood glucose 12.0 mmol/L, lactate 2.50 mmol/L, indicating severe hypoxemia and metabolic acidosis. The patient was comatose upon PICU admission, exhibiting marked respiratory distress and cyanosis of the lips. Vital signs: Temperature 39.5 °C, heart rate 183 beats/min, respiratory rate 43 breaths/min, blood pressure 104/79 mmHg, oxygen saturation 83%. Pupils were unequal in size, approximately 3.0 mm on the left and 2.0 mm on the right, with sluggish pupillary light reflexes bilaterally. Coarse breath sounds were present in both lungs with diffuse moist rales. Heart rate was rapid but regular. The abdomen was soft, without tenderness or rebound tenderness. No edema was present in the lower extremities.

The CSF routine examination results at 12:35 showed: white blood cells 3,072 × 10^6^/L, red blood cells 2.00 × 10^9^/L, and a positive Pandy test. Biochemical analysis showed total protein 3,667.6 mg/L and a glucose level of only 0.08 mmol/L, findings indicative of severe inflammatory exudation, markedly elevated protein, and profound hypoglycorrhachia. Overall, the findings were consistent with severe purulent meningoencephalitis-like changes. At this time, the primary working clinical diagnosis was “fulminant bacterial meningoencephalitis with septic shock”.

At 13:39, bedside chest radiography revealed extensive bilateral pulmonary infiltrates with localized consolidation in the right lung, consistent with severe pulmonary injury or infection. Antibiotic therapy was escalated to meropenem combined with vancomycin by intravenous infusion. Subsequent arterial blood gas analysis under high-flow oxygen therapy showed: pH 7.460, PCO_2_ 22.3 mmHg, PO_2_ 66 mmHg, BE −8.4 mmol/L, bicarbonate 15.5 mmol/L, peripheral oxygen saturation 94%, lactate 2.26 mmol/L. Repeat PCT testing in the afternoon showed an increase to 15.18 ng/mL. Coagulation function tests indicated prolonged prothrombin time (16.90 s), international normalized ratio of 1.34, decreased prothrombin activity (64.81%), fibrinogen 6.93 g/L, and D-dimer 15.05 mg/L, indicating severe systemic inflammatory response and coagulation dysfunction.

Reference ranges were based on the local laboratory standards at the testing institution and are therefore not listed individually to avoid potential misinterpretation.

## Treatment and outcomes

Due to the patient's worsening respiratory distress and hypoxemia, endotracheal intubation was performed following assessment. During intubation, a large volume of pink frothy sputum was aspirated from the endotracheal tube, indicating significant alveolar hemorrhage and/or acute pulmonary edema. Volume-controlled assist-control mechanical ventilation was subsequently initiated with the following settings: respiratory rate 18 breaths/min, tidal volume 150 mL, positive end-expiratory pressure 6 cmH_2_O, fraction of inspired oxygen 100%. Continuous infusion of midazolam and remifentanil was administered for sedation and analgesia.

Circulatory status: At 13:15, the patient’s blood pressure dropped to 80/45 mmHg. A continuous intravenous infusion of norepinephrine was initiated to maintain blood pressure. At 13:50, blood pressure decreased again, prompting the addition of intravenous dopamine. Although systolic pressure was temporarily elevated to approximately 120 mmHg, circulatory status remained extremely unstable. As the possibility of PAM was not promptly considered at that time, and pathogen results were not yet available, specific combination therapy for PAM (including amphotericin B, miconazole, azithromycin, rifampin, and fluconazole) was not administered (2, 4, 6, 11, 12).

At 15:19, monitoring indicated that the patient's heart rate suddenly dropped from 190 beats per minute to 90 beats per minute. The carotid pulse disappeared, and blood pressure could not be measured. Cardiac arrest was suspected. Cardiopulmonary resuscitation (CPR) was immediately initiated with chest compressions and intravenous administration of 0.2 mg epinephrine. By 15:23, sinus rhythm was restored, but blood pressure remained difficult to maintain. Circulation could only be barely sustained with intensive vasoactive drug support. At 15:56, the patient experienced another cardiac arrest (heart rate decreased from 190 beats/min to 110 beats/min with no palpable carotid pulse). CPR and an additional epinephrine bolus were administered, and sinus rhythm was temporarily restored at 16:02.

Despite ongoing critical care support, the patient remained in a deep coma with refractory shock. At 17:41, the patient experienced a third cardiac arrest with a sudden drop in heart rate to 55 beats per minute and loss of brachial artery pulse. Medical staff again administered prolonged advanced life support, including repeated external chest compressions, multiple injections of epinephrine (0.5 mg/dose), and 5% sodium bicarbonate to correct acidosis. At 18:10, when monitoring showed ventricular fibrillation, a 40 J biphasic non-synchronized defibrillation was administered, but spontaneous circulation could not be restored. At 18:56, the ECG continued to show a flat line. After thorough communication with the family, the patient was pronounced dead. A complete autopsy was not performed because consent was not granted by the family.

On September 5, the mNGS report for the CSF sample indicated detection of *Naegleria fowleri* sequences with high abundance (3 × 10^5^ copies/mL). No significant bacterial, viral, or other fungal/parasitic pathogen sequences were identified. Combined with the clinical presentation of fulminant meningoencephalitis and the disease course, a definitive diagnosis of PAM caused by *Naegleria fowleri* was established (7–10, 12). Blood cultures, routine CSF bacterial cultures, and available viral nucleic acid tests at the time were all negative.

The mNGS was performed on a CSF sample obtained at lumbar puncture. Approximately 2 mL of CSF was collected aseptically, placed in a sterile tube, and transported under refrigerated conditions to an independent third-party clinical laboratory. The turnaround time from sample submission to final report was approximately 3 days. CSF is the preferred specimen for CNS mNGS; samples should be handled aseptically, transported promptly under cold-chain conditions, and stored appropriately to preserve nucleic acid integrity. Sequencing reads were taxonomically classified using the laboratory's validated bioinformatics pipeline, and the report was reviewed by both the laboratory and the clinical team. The assay detected *Naegleria fowleri* with high abundance (sequence reads: 806,941) and a reported concentration of 3 × 10^5^ copies/mL.

## Discussion

This case report describes a fulminant fatal case of PAM in a 6-year-old girl, confirmed postmortem by CSF mNGS. It demonstrates several characteristic features of pediatric PAM and highlights significant diagnostic and therapeutic challenges (1).

First, the clinical presentation and CSF characteristics in this case are virtually indistinguishable from severe bacterial meningitis. Similar to previous pediatric cases, the patient presented with acute onset of high fever, headache, and vomiting, rapidly progressing to seizures, coma, and ultimately death within a very short timeframe (2, 12). CSF analysis revealed marked neutrophilic leukocytosis, markedly elevated protein levels, and severe hypoglycemia (2, 6). These features are consistent with the classic presentation of PAM while bearing a strong resemblance to purulent bacterial meningitis, making early differentiation based on etiology extremely challenging (2, 10).

Second, the normal findings on early imaging studies are consistent with previous literature reports. In the early stages of PAM, cranial computed tomography or MRI often reveals no specific abnormalities or only mild signs of cerebral edema (1, 8). The cranial MRI performed on the day of admission in this case showed no significant abnormalities, indicating that normal imaging findings cannot rule out PAM when there is strong clinical suspicion of CNS infection, especially in the early course of the disease (7, 8).

Third, this pediatric case has a significant history of freshwater exposure. Approximately 6 days prior to symptom onset, the patient had played in natural freshwater bodies. Although detailed information regarding water type, temperature, and degree of nasal immersion is lacking, the overall exposure pattern aligns with the common warm freshwater exposure scenario associated with PAM (6–9). Recent cases from China and other regions indicate that pediatric *Naegleria fowleri* infections may originate not only from lakes and rivers but also from inadequately disinfected indoor swimming pools and other artificial aquatic environments (7–9). Relevant studies demonstrate that under conditions of inadequate disinfection, elevated ambient temperatures, and aging water treatment facilities, *Naegleria fowleri* can survive in various warm freshwater sources and even some treated water systems, posing a potential threat (7).

Fourth, the diagnosis of PAM in this case relied on CSF mNGS. In recent years, mNGS has been used to confirm PAM diagnoses in multiple pediatric cases of fulminant meningoencephalitis (8). In mainland China's first reported pediatric PAM case, *Naegleria fowleri* was detected by mNGS in both CSF and blood samples, with lower sequence abundance in blood than in CSF, subsequently confirmed by polymerase chain reaction (PCR) and Sanger sequencing (11, 12). Additionally, a case report described a 6-year-old patient in Chengdu with suspected infection from indoor swimming, where CSF and blood mNGS similarly detected high-abundance *Naegleria fowleri* sequences, providing definitive etiologic evidence. In this case, CSF mNGS revealed high copy numbers of *Naegleria fowleri* sequences despite negative results from conventional CSF cultures, further highlighting the unique value of mNGS in identifying rare, unexpected, or difficult-to-detect pathogens (8–10).

Autopsy remains an important tool for postmortem evaluation in rapidly fatal central nervous system infections, as it may reveal characteristic neuropathological changes, such as hemorrhagic and necrotizing meningoencephalitis, allow direct visualization of amoebic trophozoites in brain tissue, and help exclude alternative causes of death. However, in pediatric cases, autopsy is frequently not performed because of family refusal or practical constraints. Under these circumstances, post-mortem microbiological and molecular analyses of cerebrospinal fluid and, when available, blood or tissue samples, may still provide crucial etiologic evidence and contribute to accurate diagnosis as well as public health surveillance and prevention efforts.

Regrettably, due to the extremely aggressive progression of the disease, the patient survived only approximately 11 h from admission to death. The mNGS results were reported only after death, preventing the initiation of targeted anti-amoebic combination therapy within the extremely narrow therapeutic window. In the existing literature, the few surviving cases were highly suspected of PAM in the early stages of illness and initiated combination therapy with amphotericin B (intravenous and intrathecal), miconazole, azithromycin, rifampin, fluconazole, etc., within days. This was supplemented by aggressive intracranial pressure management (including hypertonic therapy, ventriculostomy, and even hypothermia) to achieve survival. However, even with early treatment initiation, the overall survival rate for PAM remains extremely low. Therefore, enhancing the sensitivity of early recognition and exposure history inquiry is particularly critical (1–4, 11).

This case also presented with prominent pulmonary and systemic inflammatory manifestations. Following the second seizure, the patient developed hemoptysis, copious pink frothy sputum, diffuse pulmonary infiltrates, and severe hypoxemia, suggesting diffuse alveolar damage and/or hemorrhage. Together with rapidly evolving coagulation dysfunction and multiple organ dysfunction, this reflects the severity of the systemic inflammatory response syndrome and septic shock against the backdrop of fulminant central nervous system infection. This pulmonary injury may be related to both severe inflammation and increased capillary permeability, as well as high-pressure ventilation and hemodynamic fluctuations during resuscitation. Although systematic evaluations of pulmonary manifestations in PAM remain relatively limited in the current literature, case reports indicate that some patients may develop acute lung injury or pulmonary edema, warranting clinical attention (2, 4, 12).

## Clinical implications

Based on this case and previous reports, pediatric clinical practice can be summarized in four points: First, heighten vigilance. In any season, if a child or adolescent has a history of exposure to freshwater or potentially contaminated water sources (natural bodies of water, indoor pools, hot springs, religious nasal washing, nasal irrigation, etc.) and presents with acute high fever, severe headache, vomiting, seizures, and altered consciousness, accompanied by purulent inflammatory changes in the CSF, PAM should be included in the differential diagnosis. Second, expedite diagnosis. Perform lumbar puncture as early as possible under safe conditions. In addition to routine tests, concurrently conduct fresh CSF wet mount microscopy (to identify active trophozoites). Expedite submission of CSF (and blood if necessary) for mNGS or specific molecular testing to shorten the diagnostic timeline. Third, seize the therapeutic window. For clinically highly suspected cases with rapid progression, consider early initiation of anti-amoebic combination therapy centered on amphotericin B, with adjunctive agents such as azithromycin, rifampin, fluconazole, or miltefosine, alongside empirical antimicrobial treatment (13). Pediatric, infectious disease, and neurology/critical care teams should jointly assess drug availability and risk-benefit ratios. Fourth, strengthen prevention. Enhance public education and water management: avoid diving/snorkeling in potentially contaminated or inadequately disinfected water bodies; ensure standardized disinfection of swimming pools and water supply systems; use boiled and cooled or sterile water for nasal irrigation to reduce risk of nasal transmission; and bolster One Health-based environmental monitoring and interagency collaboration amid climate warming and aging infrastructure.

## Limitations

This report also has several limitations. First, due to the extremely rapid progression of the patient's condition, an environmental investigation of the exposed water source could not be conducted, making it impossible to identify the specific contaminated water body or its quality status. Second, because early imaging studies were normal and the patient's survival time was extremely short, longitudinal neuroimaging follow-up data are lacking. This makes it difficult to further characterize the evolution of PAM-related brain damage from a dynamic imaging perspective. Third, a *Naegleria fowleri*–specific PCR assay was not available or not considered while the patient was still alive, which might have enabled a more rapid etiologic diagnosis within the narrow therapeutic window. Fourth, the lack of autopsy and neuropathological examination limited pathological correlation of the clinical and molecular findings.

## Conclusion

This case presents a 6-year-old girl with PAM confirmed by CSF mNGS. The illness presented with abrupt onset and rapid progression, featuring CSF findings that closely mimicked bacterial meningitis. Early imaging studies showed no specific abnormalities, and the patient ultimately died within approximately 11 h of admission. This case highlights the importance of early consideration of PAM in children with a history of freshwater or suspected water exposure who present with rapidly progressive meningoencephalitis. Prompt lumbar puncture combined with rapid molecular diagnostics can significantly shorten the time to etiologic diagnosis. Heightened clinical vigilance and improved water environment control are essential to reducing the mortality risk associated with PAM.

## Family perspective

The parents are profoundly grieved and shocked by the disease's rapid progression and the unsuccessful resuscitation efforts. After fully understanding the purpose of case documentation and privacy protection measures, they consent to the anonymous publication of relevant clinical data. This consent aims to enhance awareness among healthcare professionals and the public regarding this rare yet fatal infection, thereby helping other families avoid similar tragedies.

## Data Availability

The original contributions presented in the study are included in the article/Supplementary Material, further inquiries can be directed to the corresponding author.
